# Increased BMPR1A Expression Enhances the Adipogenic Differentiation of Mesenchymal Stem Cells in Patients with Ankylosing Spondylitis

**DOI:** 10.1155/2019/4143167

**Published:** 2019-11-18

**Authors:** Zhenhua Liu, Peng Wang, Shuizhong Cen, Liangbin Gao, Zhongyu Xie, Xiaohua Wu, Hongjun Su, Yanfeng Wu, Huiyong Shen

**Affiliations:** ^1^Department of Orthopedics, The Eighth Affiliated Hospital of Sun Yat-sen University, 3025# Shennan Road Middle, Shenzhen 518033, China; ^2^Department of Orthopedics, Zhujiang Hospital, Southern Medical University, 253# Industrial Avenue Middle, Guangzhou 510128, China; ^3^Department of Orthopedics, Sun Yat-sen Memorial Hospital, Sun Yat-sen University, 107# Yan Jiang Road West, Guangzhou 510120, China; ^4^Center for Biotherapy, Sun Yat-sen Memorial Hospital, Sun Yat-sen University, 107# Yan Jiang Road West, Guangzhou 510120, China

## Abstract

**Objective:**

To investigate the adipogenic differentiation capacity of mesenchymal stem cells (MSCs) from ankylosing spondylitis (AS) patients and explore the mechanism of abnormal MSC adipogenesis in AS.

**Methods:**

MSCs from patients with AS (ASMSCs) and healthy donors (HDMSCs) were cultured in adipogenic differentiation medium for up to 21 days. Adipogenic differentiation was determined using oil red O (ORO) staining and quantification and was confirmed by assessing adipogenic marker expression (PPAR-*γ*, FABP4, and adiponectin). Gene expression of adipogenic markers was detected using qRT-PCR. Protein levels of adipogenic markers and signaling pathway-related molecules were assessed via Western blotting. Levels of bone morphogenetic proteins 4, 6, 7, and 9 were determined using enzyme-linked immunosorbent assays. Lentiviruses encoding short hairpin RNAs (shRNAs) were constructed to reverse abnormal bone morphogenetic protein receptor 1A (BMPR1A) expression and evaluate its role in abnormal ASMSC adipogenic differentiation. Bone marrow fat content was assessed using hematoxylin and eosin (HE) staining. BMPR1A expression in bone marrow MSCs was measured using immunofluorescence staining.

**Results:**

ASMSCs exhibited a greater adipogenic differentiation capacity than HDMSCs. During adipogenesis, ASMSCs expressed BMPR1A at higher levels, which activated the BMP-pSmad1/5/8 signaling pathway and increased adipogenesis. BMPR1A silencing using an shRNA eliminated the difference in adipogenic differentiation between HDMSCs and ASMSCs. Moreover, HE and immunofluorescence staining showed higher bone marrow fat content and BMPR1A expression in patients with AS than in healthy donors.

**Conclusion:**

Increased BMPR1A expression induces abnormal ASMSC adipogenic differentiation, potentially contributing to fat metaplasia and thus new bone formation in patients with AS.

## 1. Introduction

Ankylosing spondylitis (AS) is a chronic inflammatory and potentially disabling disease that mainly affects the axial spinal joints [1]. Structural damage in patients with AS is characterized by new bone formation and syndesmophyte development, which may lead to functional impairments and a reduced quality of life [2, 3]. Inflammation, mechanical stress, and excessive mesenchymal tissue responses have been proposed to be involved in the pathogenesis of new bone formation [4–6], but the precise mechanism underlying this process remains undefined. Recently, fat metaplasia has been suggested to be a vital process in new bone development.

Fat metaplasia, defined as focally enhanced MRI signals on T1-weighted sequences and reduced signals on short tau inversion recovery sequences, is a common MRI finding in patients with AS [7–9]. These MRI signals, which imply adipocyte accumulation [10, 11], often occur in the sacroiliac joint or vertebral corner of patients with AS. Coincidently, new bone often forms at the same sites as these signals, suggesting that fat metaplasia is an important intermediary step in new bone development [5, 7, 12]. Further studies of these MRI findings may help researchers elucidate the mechanism of new bone formation and provide insights into the pathogenesis of AS.

Mesenchymal stem cells (MSCs) are a heterogeneous population of plastic-adherent cells with a fibroblast-like morphology, immunomodulatory properties, and multilineage differentiation potential [13]. Under certain stimulation conditions, MSCs differentiate into osteocytes, chondrocytes, and adipocytes. The abnormal adipogenic differentiation of MSCs has recently been shown to contribute to fat metabolism disorders and abnormal adipocyte accumulation [14–16]. Because adipocytes in bone marrow are mainly derived from MSCs, we presume that fat metaplasia in patients with AS may be related to the adipogenic differentiation capacity of their MSCs.

Bone morphogenetic protein receptor 1A (BMPR1A), which belongs to the bone morphogenetic protein (BMP) receptor family, is a type I receptor expressed by various cells, including MSCs [17, 18]. Upon binding with BMPs to form a heterodimer, BMPR1A phosphorylates Smad1/5/8 and activates the BMP-pSmad1/5/8 signaling pathway to play a pivotal role in adipogenic differentiation [19].

In this study, we compared the adipogenic differentiation capacity of MSCs from patients with AS (ASMSCs) and healthy donors (HDMSCs) and further explored the mechanism underlying the difference. Moreover, we also assessed fat content and BMPR1A expression in the bone marrow adjacent to the sacroiliac joint. ASMSCs exhibited greater adipogenic differentiation potential because they overexpressed BMPR1A. We speculate that increased BMPR1A expression enhances the adipogenic differentiation of ASMSCs, thus contributing to fat metaplasia and new bone formation in patients with AS.

## 2. Materials and Methods

### 2.1. Ethics and Enrollment

Permission for this study was granted by the Ethics Committee of Sun Yat-sen Memorial Hospital, Sun Yat-sen University, Guangzhou, China. After obtaining written informed consent, 55 bone marrow samples were collected separately from the iliac crests of 25 patients with AS (diagnosed according to the New York modified criteria [20]) and 30 healthy donors following a standard procedure that was described in a previous study [21]. Detailed characteristics of these participants are presented in Supplemental [Supplementary-material supplementary-material-1].

### 2.2. Isolation, Expansion, and Identification of Bone Marrow-Derived MSCs

The isolation, expansion, and culture of bone marrow-derived MSCs were performed as previously described [21]. MSCs at passage 4 were used in all experiments. Flow cytometry was performed to identify the phenotypes of MSCs by detecting the expression of CD14-APC, CD29-PE, CD44-FITC, CD45-APC, CD105-FITC, and HLA-DR-PE (the antibodies were all purchased from BD, USA).

### 2.3. Adipogenic Differentiation

MSCs were seeded in 12-well plates at a density of 5 × 10^4^ cells/well in growth medium (GM) consisting of high-glucose (4500 mg/L) Dulbecco's modified Eagle's medium (DMEM, GIBCO) supplemented with 10% fetal bovine serum (FBS, Sijiqing Biological Engineering Material Company Limited, China). After the cells reached 80-90% confluence, the medium was replaced with adipogenic differentiation medium (AM) consisting of high-glucose DMEM supplemented with 10% FBS, 100 IU/mL penicillin, 100 IU/mL streptomycin, 1 *μ*M dexamethasone (Sigma-Aldrich, Germany), 200 *μ*M indomethacin (Sigma-Aldrich, Germany), 0.5 mM 3-isobutyl-1-methylxanthine (Sigma-Aldrich, Germany), and 10 *μ*g/mL insulin (Sigma-Aldrich, Germany). MSCs were cultured in AM for up to 21 days, and the medium was replaced every 3 days. MSCs cultured in GM were used as controls (0 days).

### 2.4. Cell Proliferation Assay

MSCs at the same passage were separately seeded in 96-well plates and cultured in GM or AM. Medium without cells served as a negative control. Cell proliferation was detected using a Cell Counting Kit-8 assay (Dojindo, Japan) according to the manufacturer's instructions.

### 2.5. Oil Red O (ORO) Staining and Quantification

Cells were washed with phosphate-buffered saline (PBS, BOSTER, China) and fixed with 4% formaldehyde for 20 min. Afterwards, the formaldehyde was removed, and the cells were washed with 60% isopropanol and then stained with 0.2% ORO (Sigma-Aldrich, Germany) for 30 min, as previously described [22]. The cells were then washed three times with PBS and visualized under a microscope (Olympus, Japan). Stained oil droplets were dissolved in 100% isopropanol and quantified by measuring the optical absorbance at 500 nm with a spectrophotometer (Varioskan Flash, Thermo Fisher, Germany).

### 2.6. Quantitative Real-Time Polymerase Chain Reaction (qRT-PCR)

Total RNA extraction and cDNA synthesis were performed as described in a previous study [23]. qRT-PCR was conducted in triplicate reactions for each sample with a LightCycler® 480 PCR System (Roche, Switzerland) using SYBR® Premix Ex Taq™ (Takara, Japan). The forward and reverse primers for the genes assessed using qRT-PCR are presented in Supplemental [Supplementary-material supplementary-material-1]. The relative mRNA expression of each gene was normalized to glyceraldehyde-3-phosphate dehydrogenase (GAPDH) (a housekeeping gene) using the formula 2^-*Δ*ct^.

### 2.7. Western Blot Analysis

Proteins were extracted from MSCs and quantified as previously described [21]. Equal amounts of protein extracts were denatured by boiling and then separated and transferred onto polyvinylidene fluoride (PVDF) membranes (Millipore, USA). Membranes were blocked with 5% skim milk to prevent nonspecific binding and incubated overnight at 4°C with primary antibodies against GAPDH, Smad1, pSmad1/5/8, peroxisome proliferator-activated receptor gamma (PPAR-*γ*), active *β*-catenin, *β*-catenin, CREB, p-CREB, AKT, p-AKT, ERK, p-ERK, p38 MAPK, p-p38 MAPK, JNK, p-JNK, BMP2 (all from CST, USA), fatty acid binding protein 4 (FABP4), adiponectin, BMPR1A, BMPR1B, and BMPR2 (all from Abcam, UK). Horseradish peroxidase- (HRP-) conjugated immunoglobulin IgG (Santa Cruz, USA) was used as a secondary antibody and incubated with the membranes. The immunoreactive bands were visualized using the Immobilon Western Chemiluminescent HRP Substrate (Millipore, USA).

### 2.8. Immunofluorescence Assay

Cells were washed with PBS and fixed by 4% paraformaldehyde for 30 min. Thereafter, the fixative liquid was removed by PBS and the cells were permeabilized by 0.1% Triton X-100. Then, cells were blocked with 5% skim milk and blotted using the anti-*β*-catenin in the same way as Western blot assay. Subsequently, cells were incubated with the IgG labeled anti-fluorescence. Finally, cell nucleus was stained with DAPI and visualized using laser confocal microscopy.

### 2.9. Enzyme-Linked Immunosorbent Assay (ELISA)

BMP concentrations in the cell culture supernatants were measured using BMP4 and BMP7 Quantikine ELISA kits (R&D, USA) and Human BMP6 and BMP9 ELISA kits (Sigma-Aldrich, Germany) according to the manufacturers' instructions.

### 2.10. Lentivirus Construction and Infection

Lentiviruses encoding a short hairpin RNA (shRNA) targeting BMPR1A with the sequence 5′-CATCATTTCTCGTGTTCAAGG-3′ (Lv-BMPR1A) were designed and constructed by GenePharma Co. Lentiviruses carrying the sequence 5′-TTCTCCGAACGTGTCACGT-3′ served as a negative control (Lv-NC). Lentiviruses were generated by cotransfecting 293T cells with pGLVH1/GFP/Puro (GenePharma, China) and packaging plasmids (pGag/Pol, pRev, and pVSV-G). Lentiviruses were transfected into MSCs according to the instructions in the GenePharma Recombinant Lentivirus Operation Manual (GenePharma, China). The subsequent experiments were conducted on day 10 of induction.

### 2.11. Sacroiliac Joint Biopsy and Histological Assay

Bone marrow tissues for histological assays were obtained from the vicinity of the sacroiliac joints by performing a sacroiliac joint biopsy, as detailed elsewhere [24]. Twenty-one bone marrow tissue samples were extracted after obtaining written informed consent (10 from patients with AS and 11 from healthy donors, as detailed in Supplemental [Supplementary-material supplementary-material-1]). Formalin-fixed and decalcified tissues were embedded in paraffin and cut into 4 *μ*m thick sections. For the fat content assessment, paraffin sections were stained with hematoxylin and eosin (HE). Empty holes with smooth edges were considered adipose tissues. The number and area of the adipocytes in the areas of the sampled fields were measured, and the percent adipocyte volume per marrow volume (Ad.V/Ma.V) was calculated using previously described methods [25]. For double immunofluorescence staining, paraffin sections were microwaved, blocked with 5% donkey serum, and then incubated with a primary antibody mixture containing CD105 (mouse, 1 : 50, Abcam; ab11414, UK) and BMPR1A (rabbit, 1 : 100, Abcam; ab38560, UK) at 4°C overnight. After washing, sections were incubated with a secondary antibody mixture consisting of Alexa Fluor® 488 (donkey anti-mouse IgG, 1 : 500, Invitrogen, USA) and Alexa Fluor® 546 (donkey anti-rabbit IgG, 1 : 500, Invitrogen, USA), counterstained with DAPI and visualized using laser confocal microscopy.

### 2.12. Statistical Analysis

Quantitative data are presented as the means ± standard deviations (SDs). Statistical analyses were performed using SPSS 22.0 software (Chicago). Student's *t*-test was used to assess differences between two groups, and ANOVA was conducted to assess differences among three or more groups. *P* values < 0.05 were considered statistically significant.

## 3. Results

### 3.1. HDMSCs and ASMSCs Exhibited Similarities in Morphology, Biomarker Expression, and Proliferation

**Figure 1:**
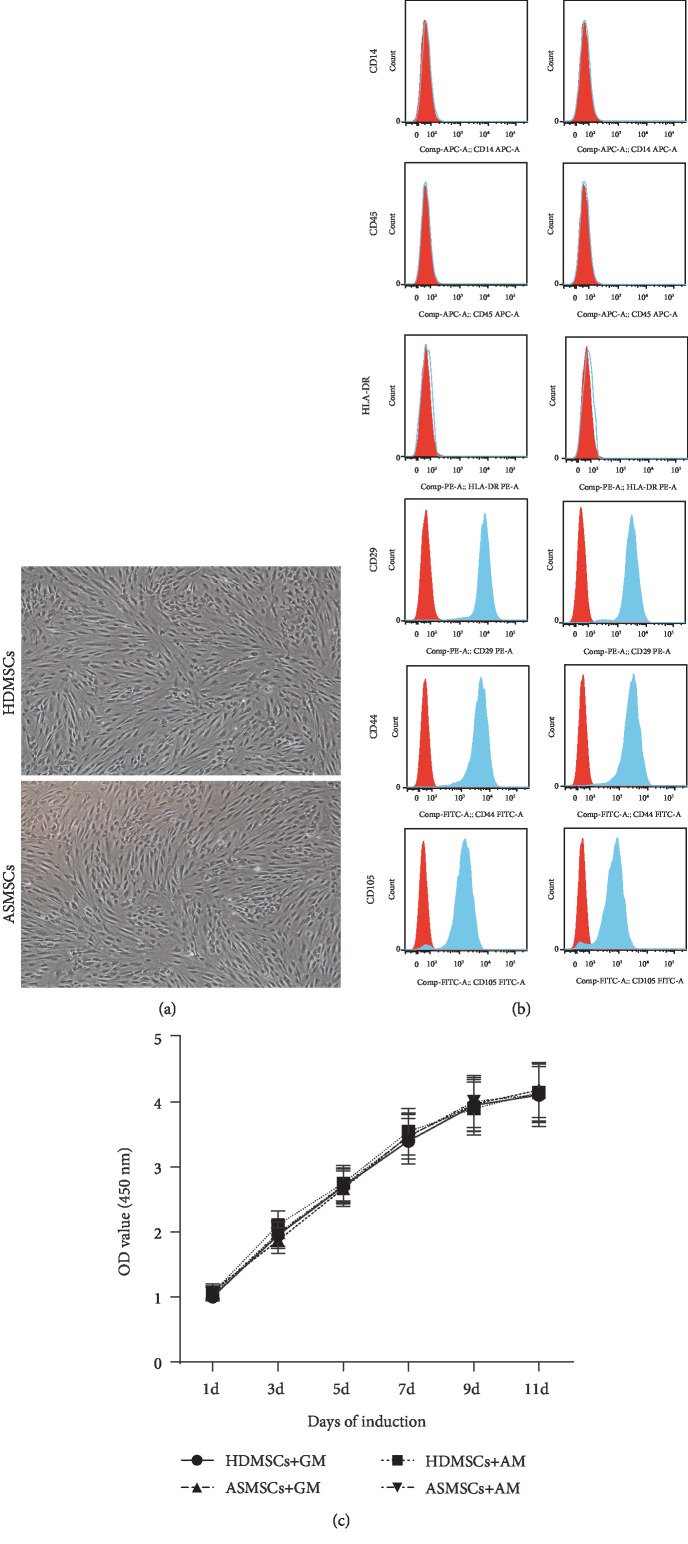
HDMSCs and ASMSCs exhibited similar morphologies, phenotypes, and proliferation rates. (a) HDMSCs and ASMSCs were both spindle-shaped, plastic-adherent cells. (b) HDMSCs (*n* = 30) and ASMSCs (*n* = 25) were negative for CD14, CD45, and HLA-DR and positive for CD29, CD44, and CD105, indicating a typical MSC phenotype. (c) HDMSCs (*n* = 30) and ASMSCs (*n* = 25) displayed equal proliferation capacities when cultured in either GM or AM from 1 to 11 days. The optical density (OD) values shown in (c) are presented as the means ± SDs.

As shown in Figure 1(a), both HDMSCs and ASMSCs were plastic-adherent cells with fibroblast-like morphologies. They expressed CD29, CD44, and CD105 but not CD14, CD45, or HLA-DR (Figure 1(b)). Moreover, HDMSCs and ASMSCs exhibited similar levels of proliferation after culture in either GM or AM for 11 days (Figure 1(c)).

### 3.2. ASMSCs Exhibited a Greater Adipogenic Differentiation Capacity than HDMSCs

**Figure 2:**
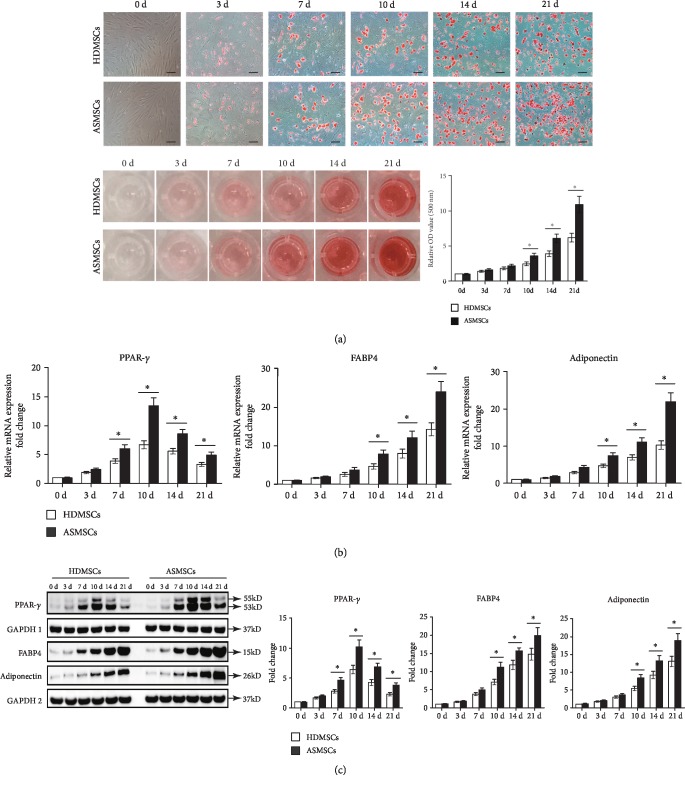
ASMSCs exhibited greater adipogenic differentiation potential than HDMSCs. The adipogenic differentiation capacities of HDMSCs (*n* = 30) and ASMSCs (*n* = 25) were determined using ORO staining and quantification and further confirmed by measuring the gene and protein expression of adipogenic markers, including PPAR-*γ*, adiponectin, and FABP4. (a) ORO staining was observed under a microscope (100x, black bars indicate 100 *μ*m), and images were captured. ASMSCs displayed darker staining than HDMSCs on days 10, 14, and 21. Consistent results were also observed for the quantification of ORO absorbance (500 nm). (b) Higher expression of PPAR-*γ* mRNA was observed in ASMSCs than in HDMSCs on days 7, 10, 14, and 21. Moreover, higher levels of adiponectin and FABP4 mRNAs were observed in ASMSCs than in HDMSCs on days 10, 14, and 21. (c) The levels of the PPAR-*γ*, adiponectin, and FABP4 proteins were consistent with their mRNA levels. Together, these results suggested that ASMSCs had greater adipogenic differentiation potential than HDMSCs. Values shown in (a–c) are presented as the means ± SDs. ^∗^*P* < 0.05 for the differences between HDMSCs and ASMSCs.

HDMSCs and ASMSCs were cultured in AM for 0-21 days. Their adipogenic differentiation capacity was assessed using ORO staining and quantification and was further confirmed by determining the expression of adipogenic markers. ORO staining increased continuously from day 0 to day 21 in both HDMSCs and ASMSCs. Compared with HDMSCs, however, ASMSCs displayed more intense staining on days 10, 14, and 21 after induction. Consistent results were also observed following the quantification of ORO staining (Figure 2(a)). In both HDMSCs and ASMSCs, the levels of the FABP4 and adiponectin mRNAs and proteins increased continuously from day 0 to day 21, but the levels of PPAR-*γ* mRNA and protein peaked on day 10 and decreased thereafter. Compared to HDMSCs, ASMSCs expressed adiponectin and FABP4 mRNAs and proteins at higher levels on days 10, 14, and 21, and they expressed PPAR-*γ* mRNA and protein at higher levels on days 7, 10, 14, and 21 (Figures 2(b) and 2(c)). Taken together, these results suggested that ASMSCs have greater adipogenic differentiation potential than HDMSCs.

### 3.3. The BMP-Smad1/5/8 Signaling Pathway Was Involved in the Enhanced Adipogenesis of ASMSCs

**Figure 3:**
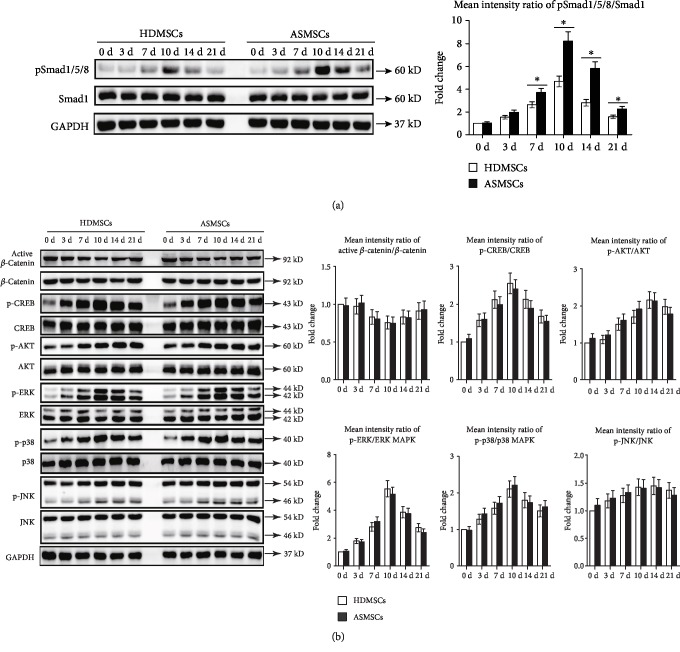
ASMSCs had higher pSmad1/5/8 expression during adipogenesis. The levels of proteins involved in adipogenic-related signaling pathways were assessed. (a) Higher levels of the pSmad1/5/8 proteins were detected in ASMSCs (*n* = 25) than in HDMSCs (*n* = 30) on days 7, 10, 14, and 21. (b) No differences in the active *β*-catenin/*β*-catenin, p-CREB/CREB, p-AKT/AKT, p-ERK/ERK, p-p38/p38, or p-JNK/JNK ratios were observed between HDMSCs and ASMSCs. Values are presented as the means ± SDs. ^∗^*P* < 0.05 for the differences between HDMSCs and ASMSCs.

Several signaling pathways related to adipogenesis were assessed to explore the mechanism underlying the enhanced adipogenic potential of ASMSCs. During adipogenic differentiation, the levels of proteins involved in all examined signaling pathways changed over time in both HDMSCs and ASMSCs. However, pSmad1/5/8/Smad1 were detected at higher levels in ASMSCs on days 7, 10, 14, and 21 than in HDMSCs, indicating the abnormal activation of BMP-pSmad1/5/8 signaling in ASMSCs during adipogenesis (Figure 3(a)). No significant differences were observed in the levels of active *β*-catenin/*β*-catenin, p-CREB/CREB, p-AKT/AKT, p-ERK/ERK, p-p38/p38, or p-JNK/JNK between HDMSCs and ASMSCs (Figure 3(b)). Additionally, the expression of *β*-catenin in the cytoplasm and nucleus was further detected by immunofluorescence assay. HDMSCs and ASMSCs had comparable *β*-catenin expression in both the cytoplasm and nucleus during adipogenesis (Supplemental [Supplementary-material supplementary-material-1]).

### 3.4. ASMSCs Expressed BMPR1A at Higher Levels during Adipogenic Differentiation

**Figure 4:**
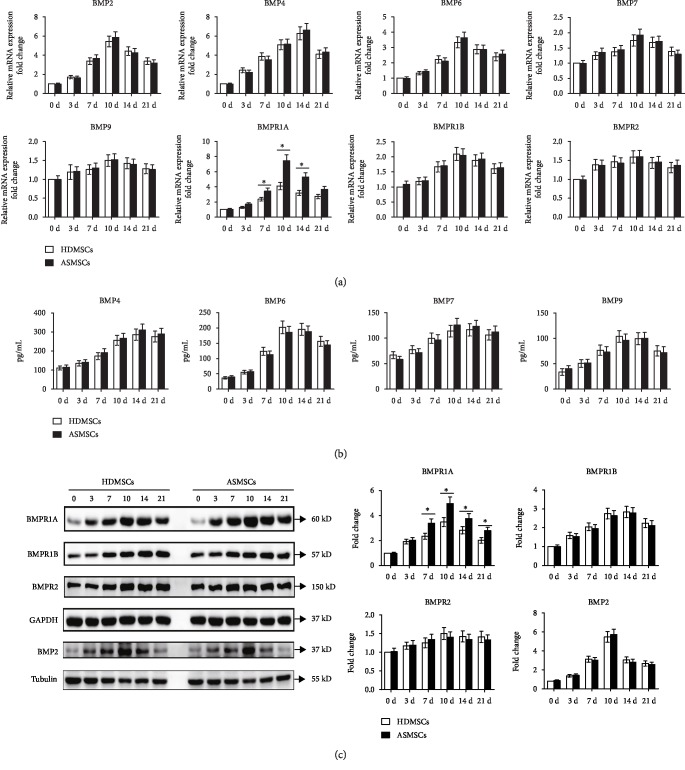
Higher BMPR1A expression was detected in ASMSCs than in HDMSCs during adipogenesis. (a) qRT-PCR was performed to detect the gene expression of BMP2, BMP4, BMP6, BMP7, BMP9, BMPR1A, BMPR1B, and BMPR2 during adipogenesis. ASMSCs (*n* = 25) expressed BMPR1A at higher levels than HDMSCs (*n* = 30) on days 7, 10, and 14. No significant difference was found between HDMSCs and ASMSCs with regard to the mRNA expression of BMP2, BMP4, BMP6, BMP7, BMP9, BMPR1B, or BMPR2. (b) ELISA was used to detect the protein expression of BMP4, BMP6, BMP7, and BMP9. The results were consistent with those of mRNA expression. (c) The protein expression of BMPR1A, BMPR1B, BMPR2, and BMP2 was determined by Western blotting. ASMSCs (*n* = 25) had higher BMPR1A protein expression than HDMSCs (*n* = 30) during adipogenic differentiation, whereas the protein expression levels of BMPR1B, BMPR2, and BMP2 were comparable between HDMSCs and ASMSCs. Values are presented as the means ± SDs. ^∗^*P* < 0.05 for the differences between HDMSCs and ASMSCs.

Levels of ligands (BMP2, 4, 6, 7, and 9) and receptors (BMPR1A, BMPR1B, and BMPR2) involved in the BMP-pSmad1/5/8 signaling pathway were detected to further explore the cause of the abnormal activation of this signaling pathway. ASMSCs exhibited higher expression of BMPR1A mRNA on days 7, 10, 14, and 21 (Figure 4(a)). Consistent results were also observed for BMPR1A protein expression (Figure 4(c)). No significant differences in the mRNA and protein expression of BMP2, BMP4, BMP6, BMP7, BMP9, BMPR1B, and BMPR2 were observed between HDMSCs and ASMSCs (Figures 4(a)–4(c)). Thus, BMPR1A was related to the abnormal activation of the BMP-Smad1/5/8 signaling pathway in ASMSCs.

### 3.5. Silencing of BMPR1A Expression Rectified the Differences in Adipogenesis between HDMSCs and ASMSCs

**Figure 5:**
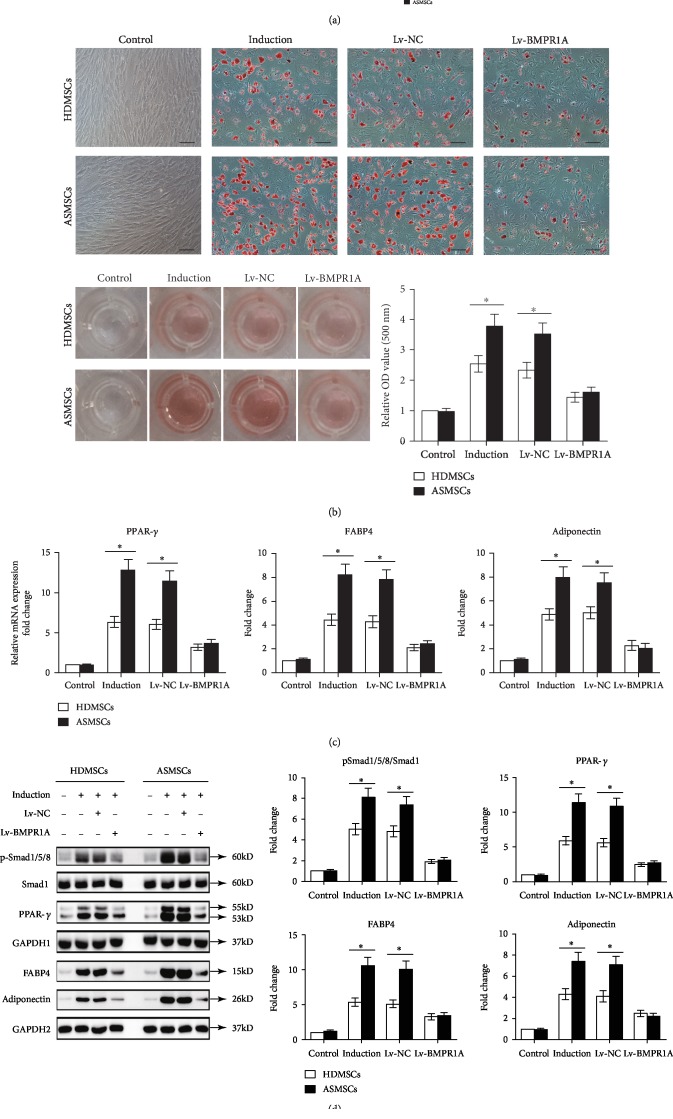
Lentiviruses encoding shRNA-BMPR1A had a greater inhibitory effect on adipogenic differentiation in ASMSCs than in HDMSCs. (a) The transfection and knockdown efficiencies were comparable between HDMSCs (*n* = 30) and ASMSCs (*n* = 25). (b) After transfection, ORO staining and ORO absorbance were decreased in both HDMSCs (*n* = 30) and ASMSCs (*n* = 25). However, Lv-BMPR1A exerted a stronger inhibitory effect on ASMSCs than on HDMSCs, leading to a comparable OD value between HDMSCs and ASMSCs. (c) Lv-BMPR1A inhibited expression of the PPAR-*γ*, FABP4, and adiponectin mRNAs in both HDMSCs (*n* = 30) and ASMSCs (*n* = 25). Lv-BMPR1A exerted a greater inhibitory effect on ASMSCs than on HDMSCs. (d) Levels of the pSmad1/5/8, PPAR-*γ*, FABP4, and adiponectin proteins in HDMSCs (*n* = 30) and ASMSCs (*n* = 25) were consistent with their gene expression levels. Values are presented as the means ± SDs. ^∗^*P* < 0.05 for the differences between HDMSCs and ASMSCs.

Lv-BMPR1A was constructed to further confirm the role that BMPR1A plays in the abnormal adipogenesis of ASMSCs. With equal transfection efficiency, Lv-BMPR1A inhibited the expression of both the BMPR1A mRNA and protein in HDMSCs and ASMSCs (Figure 5(a)). After Lv-BMPR1A transfection, ORO staining and quantification values were decreased to comparable levels in HDMSCs and ASMSCs (Figure 5(b)). Consistent results were observed for not only the expression of the PPAR-*γ*, FABP4, and adiponectin mRNAs (Figure 5(c)) but also the levels of the pSmad1/5/8, PPAR-*γ*, FABP4, and adiponectin proteins (Figure 5(d)). Although Lv-BMPR1A suppressed adipogenesis in both HDMSCs and ASMSCs, BMPR1A silencing exerted a stronger effect on BMP-pSmad1/5/8 signaling in ASMSCs than in HDMSCs and normalized the difference in adipogenic differentiation between these cell types.

### 3.6. Higher Bone Marrow BMPR1A Expression and Fat Content Were Observed in Patients with AS

**Figure 6:**
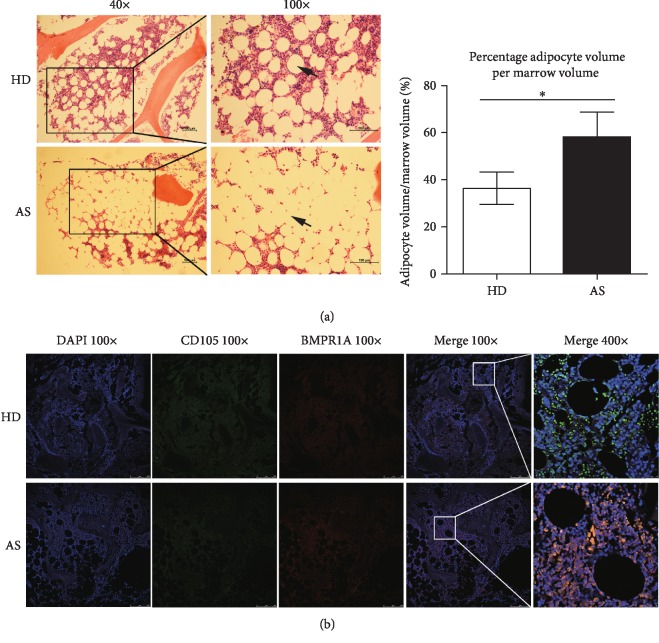
Higher fat content and BMPR1A expression were observed in bone marrow from patients with AS. (a) The bone marrow fat content in patients with AS (*n* = 10) and healthy donors (*n* = 11) was determined using HE staining (the black bars indicate 100 *μ*m). Empty holes with round, smooth edges (black arrows) were considered adipocytes. The percentage of adipocyte volume per marrow volume (Ad.V/Ma.V) was calculated and considered the bone marrow fat content. A higher bone marrow fat content was observed in patients with AS than in healthy donors. (b) Bone marrow tissues were double-stained with antibodies against BMPR1A and CD105. MSCs in the bone marrow tissues expressed both CD105 and BMPR1A, and patients with AS exhibited higher BMPR1A expression than healthy donors. Values are presented as the means ± SDs. ^∗^*P* < 0.05 for the differences between HDMSCs and ASMSCs.

Bone marrow tissues obtained from the vicinity of the sacroiliac joint were stained with HE or double-stained with antibodies against BMPR1A and CD105 (an MSC surface marker) to investigate focal BMPR1A expression and fat content in AS. Based on HE staining, the fat content in the bone marrow adjacent to the sacroiliac joint was higher in patients with AS than in healthy donors (Figure 6(a)). Furthermore, laser confocal microscopy revealed the expression of both CD105 and BMPR1A in MSCs in the bone marrow tissues, but patients with AS exhibited higher BMPR1A expression than healthy donors (Figure 6(b)).

## 4. Discussion

In the present study, ASMSCs exhibited greater adipogenic differentiation potential than HDMSCs. During adipogenesis, ASMSCs expressed BMPR1A at higher levels, which activated the BMP-pSmad1/5/8 signaling pathway and eventually contributed to abnormal adipogenic differentiation. Silencing BMPR1A expression using an shRNA had a substantial effect on BMP-pSmad1/5/8 signaling in ASMSCs and normalized the difference in adipogenesis between HDMSCs and ASMSCs. Consistent with these findings, higher BMPR1A expression and fat content in the bone marrow adjacent to the sacroiliac joint were observed in patients with AS than in healthy donors.

Fat metaplasia, also known as fat deposition, fat lesions, and fat infiltration, is a frequent MRI finding in patients with established AS. This MRI finding has attracted increasing attention due to its prognostic significance for new bone formation in patients with AS. According to a prospective study, fat metaplasia and backfill in the sacroiliac joints are key intermediaries in new bone formation [5]. Similarly, three other studies revealed strong contributions of fat metaplasia to new bone development [7, 8, 12]. However, the cause remains undefined.

Inflammation has been assumed to be the main cause of fat metaplasia for two reasons. First, fat metaplasia in patients with AS often occurs at sites where inflammation exists. Second, the severity of fat metaplasia has been reported to be related to the histopathological quantification of inflammation [26, 27]. However, according to other MRI studies, fat metaplasia may also occur and lead to new bone formation in the presence and absence of signs of inflammation [28]. These ambiguous findings prompted us to speculate that in addition to inflammation, other mechanisms may be involved in this process.

Fat transformation is closely related to the adipogenic differentiation of MSCs. Alterations in the adipogenic differentiation of MSCs contribute to abnormal fat transformation in various diseases. Increased MSC adipogenic differentiation reportedly contributes to enhanced fat metaplasia in obesity [14]. On the other hand, decreased MSC adipogenic potential contributes to the low fat mass in adult patients with idiopathic scoliosis [15]. In this study, ASMSCs displayed greater adipogenic potential than HDMSCs, potentially leading to increased adipocyte accumulation and fat metaplasia. Therefore, we consider the abnormal adipogenic differentiation of ASMSCs as a mechanism underlying fat metaplasia in patients with AS.

Curiously, this finding seems to contradict results from our previous study showing that ASMSCs possess a greater osteogenic capacity than HDMSCs [21]. Numerous studies have described an inverse relationship between adipogenesis and osteogenesis during MSC differentiation [29, 30]. The differentiation of the adipocyte lineage increases at the expense of the osteoblast lineage and vice versa [31–34]. This classic view, however, is challenged by other findings. In a specific microenvironment, both the adipogenic and osteogenic capacities of MSCs are simultaneously enhanced [15, 35, 36]. Additionally, some pleiotropic signaling pathways (such as the BMP/pSmad1/5/8 signaling pathway) have been reported to promote both osteogenesis and adipogenesis [37–40]. Moreover, specific populations of MSCs can differentiate into osteoblasts and adipocytes without affecting each other [41]. The potentiation of adipogenic differentiation does not necessarily accompany decreased osteogenesis [42, 43]. Based on these findings, the adipocyte lineage and osteoblast lineage might exhibit an independent rather than an inverse relationship in a specific milieu, which may account for the seemingly contradictory osteogenic and adipogenic differentiation capacities of ASMSCs. These seemingly contradictory results may also indicate that abnormal differentiation exists in ASMSCs and functions in different stages of pathogenic new bone formation.

The BMP/pSmad1/5/8 signaling pathway functions as one of the most important signaling pathways in the pathogenesis of AS by regulating both osteogenic and adipogenic differentiation [37, 44]. The abnormal activation of this signaling pathway may have different effects on adipogenesis and osteogenesis, depending on various factors, including the concentration and type of BMPs, the presence of extracellular and intracellular factors, the stage of differentiation, and the expression of BMP receptors [19, 38, 44, 45]. Therefore, it is interesting but not surprising that when activated by increased BMP2 expression, this signaling pathway promoted enhanced osteogenesis of ASMSCs in our previous study [21], whereas when activated by increased BMPR1A, this signaling pathway contributed to enhanced adipogenesis of ASMSCs in the present study. Discerning the underlying mechanism of the abnormal expression of BMP2 and BMPR1A may help to explain this interesting phenomenon. Recent studies have suggested that single nucleotide polymorphisms (SNPs) may play a role in the abnormal expression of these factors and in the pathogenesis of AS. It has been reported that rs3178250, a SNP located in the 3′ ultraconserved region of BMP2, is correlated with AS by potentially modulating the translation and protein expression of BMP2 [46, 47]. Similarly, BMPR1A SNPs were demonstrated to be correlated with obesity-related diseases by increasing the mRNA expression of BMPR1A [48]. Therefore, it is reasonable to presume that the increased BMPR1A expression in the present study may also be correlated with BMPR1A SNPs. Future studies should shed more light on the relationship between BMPR1A SNPs and AS.

The histopathology of fat metaplasia remains controversial. With enhanced signals on T1-weighted sequences and reduced signals on short tau inversion recovery sequences, fat metaplasia often indicates increased fat replacement based on adipocyte accumulation [11]. However, in some cases, this MRI finding may also reflect lipid accumulation in other cells [49], such as macrophages [50], suggesting that this MRI finding does not necessarily imply adipocyte accumulation in patients with AS. In support of this hypothesis, a recent study suggested that the fat content in the bone marrow adjacent to the zygapophyseal joint was not increased in patients with AS [51]. However, in the present study, a higher fat content in the bone marrow adjacent to the sacroiliac joint was observed in patients with AS than in healthy donors. Age bias may account for this difference. Patients with AS were significantly younger than healthy donors in the former study, but the ages of these groups were comparable in our study. Considering that the amount of adipose tissue in the bone marrow increases with age [52], the bone marrow fat content in patients with AS might have been underestimated in the previous study. Additionally, a recent study on the vertebral edge of AS further demonstrated that the histological composition of this MRI finding is adipocyte accumulation [53]. Therefore, we believe that this MRI finding in patients with AS refers to increased fat replacement based on adipocyte accumulation. However, further studies are still needed because the sample size was not sufficient in either the previous studies or our present study.

In the present study, increased BMPR1A expression induced abnormal adipogenic differentiation of ASMSCs. These results may improve our understanding of the mechanism of fat metaplasia and provide insights into the pathogenesis of new bone formation. Moreover, approaches that manipulate BMPR1A expression may be a novel therapeutic strategy to rectify the abnormal adipogenic differentiation of ASMSCs, fat metaplasia, and subsequent new bone formation. However, some limitations cannot be ignored in this study. How does the focal microenvironment in vivo affect MSC differentiation? What is the precise cause of increased BMPR1A expression? How does abnormal adipogenesis contribute to new bone formation? Why does fat metaplasia result in new bone formation in patients with AS but develop into bone erosion in patients with rheumatoid arthritis? Future studies should answer these questions to illuminate the mechanisms of fat metaplasia and new bone development in patients with AS.

## Data Availability

The data used to support the findings of the study entitled “Increased BMPR1A Expression Enhances the Adipogenic Differentiation of Mesenchymal Stem Cells in Patients with Ankylosing Spondylitis (4143167)” are included within the article and the supplementary information files.

## Supplementary Materials

Supplementary Materials Supplemental Figure 1: HDMSCs and ASMSCs had comparable *β*-catenin expression in both the cytoplasm and nucleus during adipogenesis. Supplemental Table 1: characteristics of the study subjects who donated bone marrow samples. Supplemental Table 2: primers used for qRT-PCR. Supplemental Table 3: characteristics of the study subjects who donated bone marrow tissues.
