# Supplementary material for: Increased BMPR1A Expression Enhances the Adipogenic Differentiation of Mesenchymal Stem Cells in Patients with Ankylosing Spondylitis
Source: Stem Cells Int. 2019 Nov 18;2019:4143167. doi: 10.1155/2019/4143167 (PMC6885782; doi:10.1155/2019/4143167)
Supplement: Supplementary Materials — Supplemental Figure 1: HDMSCs and ASMSCs had comparable β-catenin expression in both the cytoplasm and nucleus during adipogenesis. Supplemental Table 1: characteristics of the study subjects who donated bone marrow samples. Supplemental Table 2: primers used for qRT-PCR. Supplemental Table 3: characteristics of the study subjects who donated bone marrow tissues. [file 4143167.f1.pdf]

Supplemental Figure 1. HDMSCs and ASMSCs had comparable β-catenin expression in both the cytoplasm and nucleus during adipogenesis

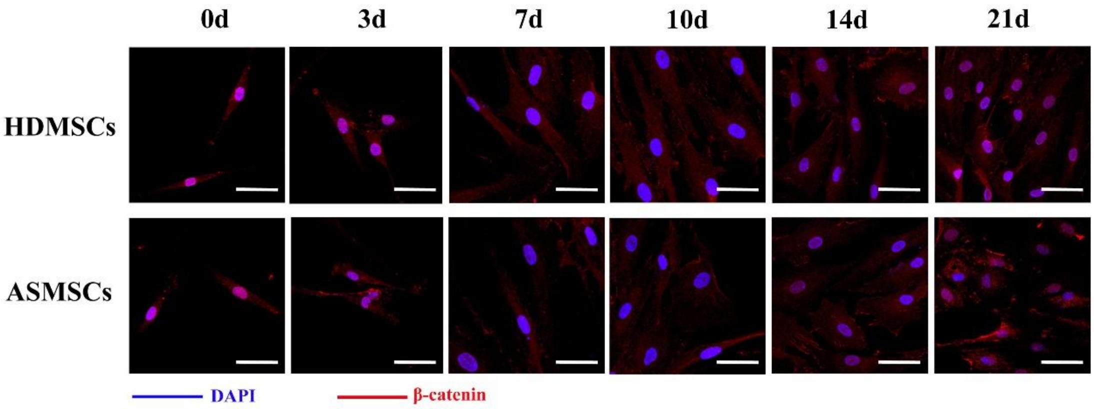

Expression of β-catenin in both the cytoplasm and nucleus during adipogenesis were determined by immunofluorescence assay (200×, the white bars indicate 50 μm). Nucleus was stained with DAPI (blue), whereas the expression of β-catenin (red) in both the cytoplasm and nucleus were detected by anti-β-catenin. HDMSCs (n = 6) and ASMSCs (n = 6) had comparable β-catenin expression in both the cytoplasm and nucleus during adipogenesis.

Supplemental Table 1. Characteristics of the study subjects who donated bone marrow samples

	Healthy donors	Patients with AS
**Number**	30	25
**Age (years)**	30.5 ± 10.2	32.3 ± 13.1
**Males, No. (%)**	19 (63.3%)	17 (68.0%)
**HLA-B27-positive, No. (%)**	0	23 (92.0%)*
**Disease duration (years)**	NA	6.94 ± 6.3
**CRP (mg/L)**	3.5 ± 1.4	18.8 ± 13.3*
**ESR (mm/h)**	10.5 ± 3.6	28.2 ± 14.8*
**BASDAI**	0.92 ± 0.88	4.72 ± 1.03*

Means ± SDs. AS, ankylosing spondylitis; HLA-B27, human leukocyte antigen B27; CRP, C-reactive protein; ESR, erythrocyte sedimentation rate; BASDAI, Bath ankylosing spondylitis disease activity index; * P< 0.05 compared to healthy donors.

Supplemental Table 2. Primers used for qRT-PCR

Gene	Accession No.	Forward primer (5'-3')	Reverse primer (5'-3')	Product size (bp)
**GAPDH**	NM_001256799	GGAGCGAGATCCCTCCAAAAT	GGCTGTTGTCATACTTCTCATGG	197
**PPAR–γ**	NM_138711	GGGATCAGCTCCGTGGATCT	TGCACTTTGGTACTCTTGAAGTT	186
**FABP4**	NM_001442	ACTGGGCCAGGAATTTGACG	CTCGTGGAAGTGACGCCTT	183
**Adiponectin**	NM_001177800	AACATGCCCATTCGCTTTACC	TAGGCAAAGTAGTACAGCCCA	107
**BMPR1A**	NM_004329	AGATGACCAGGGAGAAACCAC	CAACATTCTATTGTCCGGCGTA	111
**BMPR1B**	NM_001256792	CTTTTGCGAAGTGCAGGAAAAT	TGTTGACTGAGTCTTCTGGACAA	130
**BMPR2**	NM_001204	CACTCAGTCCACCTCATTCATTT	TTGTTTACGGTCTCCTGTCAAC	131
**BMP2**	NM_001200	GCCAAACACAAACAGCGGAA	GGGAGCCACAATCCAGTCAT	102
**BMP4**	NM_001202	ATGATTCCTGGTAACCGAATGC	CCCCGTCTCAGGTATCAAACT	93
**BMP6**	NM_001718	TGTTGGACACCCGTGTAGTAT	AACCCACAGATTGCTAGTGGC	80
**BMP7**	NM_001719	GGAACGCTTCGACAATGAGAC	GCAGGAAGAGATCCGATTCCC	86
**BMP9**	NM_016204.2	GCTTCAGCATGGAAGATGCC	CCACGCTTCCTTTCAGGTCA	180

GAPDH, glyceraldehyde-3-phosphate dehydrogenase; **PPAR-γ**, peroxisome proliferator-activated receptor gamma; **FABP4**, fatty acid binding protein 4; **BMPR**, bone morphogenetic protein receptor; **BMP**, bone morphogenetic protein.

Supplemental Table 3. Characteristics of the study subjects who donated bone marrow tissues

	**Healthy donors**	**Patients with AS**
**Number**	11	10
**Age (years)**	28.9 ± 11.0	29.7 ± 12.5
**Males, No. (%)**	8 (72.7%)	8 (80.0%)
**HLA-B27-positive, No. (%)**	0	9 (90.0%)*
**Disease duration (years)**	NA	4.84 ± 3.2
**CRP (mg/L)**	3.3 ± 1.5	18.4 ± 11.2*
**ESR (mm/h)**	12.8 ± 4.2	31.2 ± 15.1*
**BASDAI**	0.95 ± 0.87	4.67±1.02*

Means ± SDs. AS, ankylosing spondylitis; HLA-B27, human leukocyte antigen B27; CRP, C-reactive protein; ESR, erythrocyte sedimentation rate; BASDAI, Bath ankylosing spondylitis disease activity index; * $P < 0.05$ compared to healthy donors.
